# Antihypertensive Therapy and Survival Outcomes in Patients with Renal Cell Carcinoma: A Retrospective Cohort Study

**DOI:** 10.3390/jcm15082995

**Published:** 2026-04-15

**Authors:** Sara İleri, Mehmet Emin Yılmaz, Öztürk Ateş

**Affiliations:** 1 Nephrology Department, Gülhane Training and Research Hospital, 06010 Ankara, Türkiye; 2 Oncology Department, Dr. Abdurrahman Yurtaslan Oncology Training and Research Hospital, 06200 Ankara, Türkiye; aslanim6606@gmail.com (M.E.Y.); dr.ozturkates@yahoo.com (Ö.A.)

**Keywords:** renal cell carcinoma, hypertension, antihypertensive therapy, prognosis

## Abstract

**Background/Objectives:** Hypertension is a recognized risk factor for renal cell carcinoma; however, the impact of antihypertensive therapy AHT on clinical outcomes in patients with established RCC remains insufficiently understood. This study aimed to evaluate the association between antihypertensive therapy use and clinical outcomes, particularly overall survival, in patients diagnosed with renal cell carcinoma. **Methods:** This retrospective cohort study included 100 patients with renal cell carcinoma treated at a single center. Antihypertensive medications were evaluated according to both the presence of therapy and the number of agents used. Overall survival was defined as the time from renal cell carcinoma diagnosis to death from any cause or last follow-up. Survival outcomes were analyzed using Kaplan–Meier methods and Cox proportional hazards regression. **Results:** Hypertension was present in 66% of patients, all of whom were receiving antihypertensive therapy. Kaplan–Meier analysis demonstrated significantly longer overall survival among AHT users compared with non-users (log-rank *p* = 0.0007), with median overall survival 17.1 months in the non-AHT group and 112.2 months in the AHT group. After propensity score adjustment, antihypertensive therapy remained associated with improved survival (HR 0.35, 95% CI 0.13–0.94, *p* = 0.036). Antihypertensive therapy was not associated with tumor size, stage, or metastasis at diagnosis. **Conclusions:** Antihypertensive therapy use at diagnosis was associated with improved overall survival in patients with renal cell carcinoma. This association was not observed for tumor size, stage at diagnosis, or metastasis status, although nodal involvement was less frequent among patients receiving antihypertensive therapy. Because antihypertensive therapy exposure overlapped with hypertension status and baseline differences existed between groups, the observed survival advantage should be interpreted cautiously and considered hypothesis-generating rather than evidence of a causal relationship.

## 1. Introduction

Renal cell carcinoma (RCC) is the most common primary malignancy of the kidney, accounting for more than 90% of kidney cancers and approximately 3% of all adult malignancies. It occurs predominantly in a sporadic form (~96%) and less commonly as a familial condition (~4%), and consists of several genetically distinct subtypes. RCC most frequently affects individuals between 60 and 70 years of age and demonstrates an approximately 2:1 male-to-female ratio. Despite increasing incidence, mortality rates have declined by approximately 1% annually since 2008 [1].

Prognosis in RCC depends strongly on disease stage at diagnosis. The five-year survival rate approaches 93% in patients with early-stage disease but decreases to approximately 12% in patients with metastatic RCC, many of whom require systemic therapy [2].

RCC often remains clinically silent until advanced stages, and more than 50% of tumors are detected incidentally during imaging performed for unrelated conditions. Only 10–15% of patients present with the classic triad of flank pain, hematuria, and a palpable flank mass. Historically, painless hematuria was reported in more than 60% of cases at presentation. Additional manifestations may include fatigue, weight loss, fever, night sweats, malaise, hypertension, and anemia. Varicocele may also occur as a result of tumor extension into the renal vein and inferior vena cava, leading to obstruction of the testicular vein [3]. Major histological subtypes include clear cell RCC (CCRCC), papillary RCC (PRCC), chromophobe RCC, collecting duct carcinoma, and unclassified RCC. Clear cell RCC represents the most common subtype, accounting for approximately 70–80% of cases [2].

Hypertension (HTN) is a major global health concern affecting approximately 34% of adults worldwide, and its prevalence continues to increase [4]. Accumulating evidence identifies hypertension as a significant and dose-dependent risk factor for RCC development [5,6,7,8]. A meta-analysis demonstrated that each 10 mmHg increase in systolic and diastolic blood pressure was associated with approximately 10% and 20% increases in kidney cancer risk, respectively [9,10]. Conversely, risk reduction observed after blood pressure control suggests that hypertension management may contribute to RCC prevention [10].

Although the association between hypertension and RCC incidence has been extensively investigated, considerably less is known about the prognostic significance of antihypertensive therapy in patients with established RCC.

The relationship between antihypertensive therapy and RCC risk is complex. Several studies suggest that associations between antihypertensive medications and RCC risk may reflect underlying hypertension or protopathic bias—where hypertension represents an early manifestation of occult kidney disease—rather than a direct pharmacologic effect of treatment [7,11]. Indeed, an increased RCC risk (relative risk [RR] = 1.6) has been observed among antihypertensive medication users compared with the general population, although a causal relationship is generally considered unlikely [12].

Experimental evidence suggests that blockade of the renin–angiotensin system (RAS) suppresses tumor growth, metastasis, and angiogenesis in several malignancy models. Retrospective clinical studies similarly indicate that long-term angiotensin-converting enzyme inhibitor use may influence cancer progression [13].

Given the complex interplay between hypertension, antihypertensive therapy, and RCC risk and prognosis, the present study aimed to evaluate the association between antihypertensive therapy use at diagnosis and overall survival in patients with RCC. Secondary objectives included examining whether the number of antihypertensive agents and hypertension status were associated with tumor characteristics and clinical outcomes.

## 2. Materials and Methods

This retrospective single-center cohort study included patients diagnosed RCC between January 2003 and December 2024 at Ankara Dr. Abdurrahman Yurtaslan Oncology Training and Research Hospital. AI tool was used for English editing.

A total of 100 patients with histologically confirmed RCC were included in the analysis. One patient had incomplete baseline covariate data and was therefore excluded from Table 1 comparisons but retained in descriptive cohort summaries.

Clinical data collected at diagnosis included demographic characteristics, lifestyle factors, and disease-related variables, including hypertension status, presence of additional comorbidities, RCC subtype (clear cell, chromophobe, papillary, sarcomatoid), International Metastatic Renal Cell Carcinoma Database Consortium (IMDC) score, stage at diagnosis (I–IV), tumor size (mm), nodal involvement (N), and metastasis status (M).

Stage information was unavailable for four patients; therefore, stage distributions were reported for 95 patients with complete staging data.

A substantial proportion of patients presented with advanced disease at diagnosis (stage IV: 65.7%), consistent with the tertiary referral nature of the study center.

Patients were eligible for inclusion if they were aged ≥18 years, had histologically confirmed RCC, had available baseline clinical and treatment data, and had documented antihypertensive medication status at the time of diagnosis. Patients were excluded if survival follow-up data were unavailable, antihypertensive treatment information was incomplete, or kidney malignancies other than RCC were present.

Patients were identified through institutional oncology registry records and electronic medical charts. Consecutive eligible patients meeting inclusion criteria during the study period were included to reduce selection bias.

Antihypertensive therapy (AHT) exposure was defined as treatment present at the time of diagnosis. Antihypertensive medication use was evaluated both as a binary variable (presence vs. absence of therapy) and according to the number of agents prescribed (one, two, three, or four or more). Patient outcomes were defined according to final survival status (alive or deceased).

For comparisons between two groups (e.g., AHT users vs. non-users), independent-samples *t*-tests were used for continuous variables (e.g., age, tumor size), while χ^2^ tests or Fisher’s exact tests were used for categorical variables (e.g., sex, alcohol use, smoking status, comorbidities, RCC subtype, surgical treatment status, nodal involvement, metastasis status, treatment line receipt, and survival status), as appropriate.

Variables included in the multivariable Cox proportional hazards regression model were selected a priori based on established clinical relevance and previously reported prognostic importance in RCC, including age, stage at diagnosis, IMDC risk score, surgical treatment status, nodal involvement, and metastasis status. As a sensitivity analysis, the multivariable Cox regression model was additionally adjusted for smoking status and comorbidity burden to evaluate the robustness of the association between antihypertensive therapy use and overall survival.

The proportional hazards assumption was assessed using Schoenfeld residuals, and no significant violations were detected.

For comparisons involving more than two groups (e.g., categories of antihypertensive treatment number), one-way analysis of variance (ANOVA) was used for continuous variables. When ANOVA identified statistically significant differences, appropriate post hoc pairwise comparisons were performed using Tukey’s HSD test. A two-sided *p* value < 0.05 was considered statistically significant for all analyses.

In addition to evaluating antihypertensive treatment use as a binary variable and by number of agents, antihypertensive medications were categorized according to pharmacologic class, including renin–angiotensin system inhibitors (ACE inhibitors or angiotensin receptor blockers), calcium channel blockers, beta-blockers, and diuretics. Descriptive analyses were performed to evaluate the distribution of these classes within the cohort. Due to the limited sample size, survival comparisons between individual drug classes were considered exploratory.

Survival analyses were conducted in 97 patients with complete time-to-event information. Although vital status was available for the entire cohort, three patients lacked complete date information required for survival time calculation and were therefore excluded from time-to-event analyses but retained in descriptive analyses. No imputation procedures were applied due to the limited number of missing observations.

Overall survival (OS) was defined as the time from RCC diagnosis to death from any cause or last follow-up. Survival time was calculated using the diagnosis date as the starting point and the date of death or last recorded follow-up as the endpoint. Patients alive at last follow-up were censored.

Kaplan–Meier survival analysis was used to estimate survival distributions. Survival curves were generated to evaluate overall survival and to compare survival between antihypertensive therapy users and non-users. Differences between groups were assessed using the log-rank test, and survival time was expressed in months.

In multivariable Cox proportional hazards regression analysis, stage at diagnosis was excluded from the final model due to collinearity with metastasis status and IMDC risk score. The final model included antihypertensive therapy use, age, IMDC score, surgical treatment status, nodal involvement, and metastasis status.

To further reduce confounding by indication, a propensity score analysis was performed. Propensity scores representing the probability of receiving antihypertensive therapy were estimated using logistic regression including age, smoking status, comorbidity burden, IMDC score, nodal involvement, metastasis status, and surgery. A propensity score-adjusted Cox proportional hazards regression model was then used to evaluate the association between antihypertensive therapy use and overall survival. As an additional sensitivity analysis, a Cox model stratified by propensity score quintiles was also performed.

## 3. Results

A total of 100 patients with RCC were included in the study. Baseline characteristics were available for 99 patients and are summarized in Table 1. The majority of patients were male (72.7%), with a mean age of 59.5 ± 10.9 years. Hypertension was present in 66% of the cohort, and 79% of patients had at least one additional comorbidity. Smoking was reported in 61% of patients, whereas alcohol use was uncommon (4.0%).

Clear cell RCC was the predominant histological subtype (82.8%). A substantial proportion of patients presented with advanced disease at diagnosis, with 65.7% classified as stage IV and 64.0% demonstrating distant metastases. Nodal involvement was observed in 40.0% of patients. The mean tumor size at diagnosis was 79.6 ± 34.6 mm.

Surgical treatment was performed in 53.0% of patients. Nearly all patients received first-line systemic therapy (99.0%), whereas subsequent treatment lines were administered less frequently: 72.7% received second-line therapy, 47.5% received third-line therapy, and 39.4% received fourth-line therapy.

Among patients with hypertension, all were receiving antihypertensive therapy. When patients were stratified according to AHT exposure status, those receiving antihypertensive therapy were significantly older (61.9 ± 9.2 vs. 54.8 ± 12.3 years, *p* = 0.001) and had a higher prevalence of additional comorbidities (93.8% vs. 52.9%, *p* = 0.001). They also had lower smoking rates (50.8% vs. 82.4%, *p* = 0.002) and a lower frequency of nodal involvement at diagnosis (30.8% vs. 55.9%, *p* = 0.020). Surgical treatment was more frequently performed in patients receiving antihypertensive therapy (63.1% vs. 35.3%, *p* = 0.011), and these patients were more likely to receive third-line systemic therapy (58.5% vs. 26.5%, *p* = 0.032).

No statistically significant differences were observed between AHT users and non-users with respect to sex, alcohol use, histological subtype, stage at diagnosis, tumor size, metastasis status, receipt of second-line or fourth-line systemic therapy, or survival status at last follow-up.

At the last recorded follow-up, 62 deaths were documented in the overall cohort. Time-to-event analyses were restricted to 97 patients with complete survival-time data, among whom 33 deaths were observed. The remaining deaths lacked complete date information required for survival time calculation and were therefore excluded from Kaplan–Meier and Cox regression analyses. Baseline characteristics of excluded patients did not differ substantially from those included.

Of the 33 deaths observed among patients with complete survival-time data, two occurred outside the predefined time window used for Kaplan–Meier estimation and were therefore not included in the group-specific survival summaries presented in Table 2.

**Figure 1:**
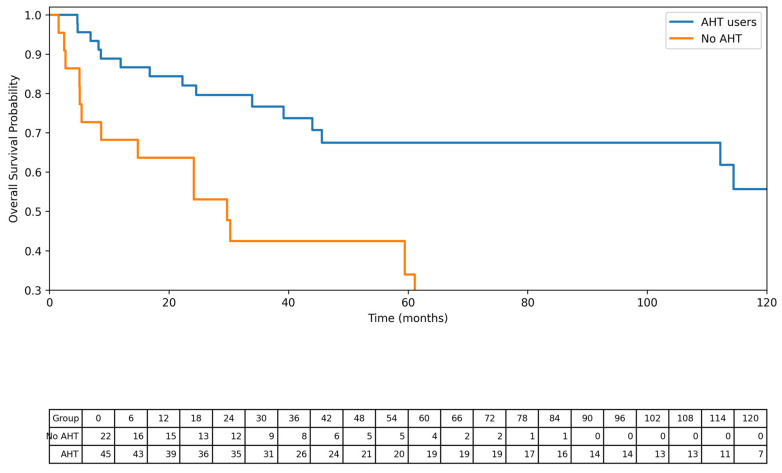
Kaplan–Meier overall survival according to antihypertensive therapy use. Kaplan–Meier curves showing overall survival stratified by antihypertensive therapy (AHT) use at the time of renal cell carcinoma diagnosis. Overall survival was defined as the time from diagnosis to death from any cause or last follow-up. Patients alive at last follow-up were censored. Kaplan–Meier survival analysis demonstrated significantly longer overall survival among patients receiving antihypertensive therapy compared with non-users (log-rank *p* < 0.001). Median overall survival was 17.1 months in the non-AHT group and 112.2 months in the AHT group. Numbers at risk at 6-month intervals are displayed below the x-axis.

Kaplan–Meier survival analysis demonstrated significantly longer overall survival among patients receiving antihypertensive therapy compared with non-users (log-rank *p* < 0.001). Median overall survival was 17.1 months in the non-AHT group and 112.2 months in the AHT group. (Figure 1)

In multivariable Cox proportional hazards regression analysis including antihypertensive therapy use, age, IMDC score, surgical treatment status, nodal involvement, and metastasis status, antihypertensive therapy remained independently associated with improved overall survival (HR 0.26, 95% CI 0.13–0.50, *p* < 0.001). Higher IMDC score was independently associated with worse survival (HR 1.92, 95% CI 1.41–2.61, *p* < 0.001), whereas surgical treatment was associated with improved survival (HR 0.16, 95% CI 0.06–0.45, *p* = 0.001). Age, nodal involvement, and metastasis status were not independently associated with overall survival after adjustment. Stage at diagnosis was not included in the final model because of conceptual overlap with metastasis status and IMDC risk score (Table 3).

In the propensity score-adjusted Cox regression model, antihypertensive therapy remained associated with improved overall survival (HR 0.35, 95% CI 0.13–0.94, *p* = 0.036). In a sensitivity analysis using Cox regression stratified by propensity score quintiles, the association remained borderline significant (HR 0.32, 95% CI 0.10–1.00, *p* = 0.049).

Additional sensitivity analyses adjusting for smoking status and comorbidity burden yielded results consistent with the primary multivariable Cox regression model, supporting the stability of the observed association between antihypertensive therapy use and improved overall survival.

Metastasis status was not independently associated with survival after adjustment for IMDC score, likely reflecting shared prognostic information between these variables.

## 4. Discussion

This retrospective study evaluated the association between AHT use and survival outcomes in patients with RCC. Hypertension was highly prevalent in this cohort (66%), consistent with previous evidence identifying hypertension as an important risk factor for RCC development [4,5,9].

Kaplan–Meier survival analysis demonstrated significantly longer overall survival among patients receiving antihypertensive therapy compared with non-users. In multivariable Cox proportional hazards regression analysis adjusting for established prognostic factors—including age, stage at diagnosis, IMDC risk score, surgical treatment status, and metastasis status—AHT remained independently associated with a reduced risk of death. These findings suggest that AHT use was associated with improved survival outcomes in this cohort.

However, this association should be interpreted cautiously. Patients receiving antihypertensive therapy differed from non-users in several baseline characteristics. Specifically, they were older, had a higher prevalence of comorbidities, had lower rates of nodal involvement at diagnosis, and were more likely to undergo surgical treatment. In addition, patients receiving AHT were more likely to receive later lines of systemic therapy, including third-line treatment. These differences may reflect variations in clinical surveillance, treatment selection, or overall fitness for therapy rather than a direct protective effect of antihypertensive medications themselves.

Residual confounding and selection bias cannot be excluded because of the retrospective design of the study. Additional sensitivity analyses adjusting for smoking status and comorbidity burden yielded results consistent with the primary multivariable Cox regression model, supporting the robustness of the observed association. Propensity score matching was not performed due to the limited sample size; instead, propensity score-adjusted and stratified Cox regression models were applied to reduce confounding by indication. Nevertheless, residual confounding related to treatment eligibility and baseline disease characteristics may still partially explain the observed survival advantage.

The literature consistently identifies hypertension as an independent risk factor for RCC incidence. Several studies have demonstrated a dose-dependent relationship between increasing systolic and diastolic blood pressure and RCC risk, and some evidence suggests that effective blood pressure control may reduce the likelihood of RCC development [4,9,10]. Similarly, a dose–response relationship between increasing systolic and diastolic blood pressure and renal cell carcinoma risk has also been confirmed in additional analyses [10].

In contrast, the relationship between antihypertensive medications and RCC risk remains controversial and is often complicated by underlying hypertension and protopathic bias [7,14]. Some meta-analyses based on randomized controlled trials have not demonstrated a consistent association between antihypertensive therapy and overall cancer risk [11]. These findings are consistent with the present study, in which antihypertensive therapy was not associated with tumor size, stage at diagnosis, nodal involvement, or metastasis status.

Nevertheless, several observational studies have reported conflicting results. Fryzek et al. reported an increased RCC risk among antihypertensive medication users compared with the general population (RR = 1.6) [8]. Other population-based cohort studies have also suggested that kidney cancer risk increases with both blood pressure level and the number of antihypertensive drug classes used [15,16]. These discrepancies highlight the difficulty of separating the effects of hypertension itself from those of antihypertensive medications in observational studies.

Evidence regarding individual antihypertensive drug classes remains inconsistent. Diuretics have historically been implicated in RCC risk and have been reported to increase RCC incidence in some meta-analyses by up to 34% As the duration of diuretic use increases, the risk of kidney cancer has also been observed to rise. Analyses indicate that each additional year of diuretic use is associated with approximately a 2% increase in the incidence of kidney cancer. In particular, long-term use of 16 years or more has been linked to a higher risk [16,17,18,19]. Some studies suggest that diuretic use may be more strongly associated with specific RCC subtypes rather than with overall RCC risk. For example, long-term diuretic use has been reported to significantly increase the risk of papillary RCC (OR = 3.1) [20]. In addition, a positive association has been observed between diuretic use and cases of clear cell RCC without VHL mutations (RR = 2.11) [18,19,20,21].

One of the ongoing scientific debates concerns whether the observed risk is attributable to diuretic use itself or to the underlying hypertension being treated. Some studies have reported that the association between diuretic use and cancer weakens or disappears after adjustment for hypertension, whereas others suggest that diuretics may confer an independent risk beyond established factors such as hypertension and smoking [19,22].

Calcium channel blockers have also been investigated for potential carcinogenic mechanisms, including possible inhibition of apoptosis [22,23]. Similarly, beta-blockers have been associated with RCC risk in some epidemiological studies, although these associations appear to weaken over time [17]. In contrast, renin–angiotensin system inhibitors, including angiotensin-converting enzyme inhibitors and angiotensin receptor blockers, have been suggested to have neutral or potentially protective effects in some studies

These findings suggest that, rather than the medications themselves being directly carcinogenic, hypertension may act as an independent risk factor for kidney cancer. In many studies, the observed increase in risk associated with these medications may primarily reflect the effects of chronic exposure to elevated blood pressure or shared risk factors related to hypertension, such as obesity and smoking.

However, according to research presented by Dr. Rana McKay of Dana-Farber Cancer Institute at the 2014 Genitourinary Cancers Symposium it has been shown that patients with advanced RCC receiving angiotensin-converting enzyme inhibitors (ACE-Is) had a mean survival of 26.68 months, compared with 17.05 months in those not receiving these agents. This suggests that ACE-I—especially lisinopril, kaptopril and angiotensinogen receptor blockers (ARBs) such as losartan—use may be associated with an extension of survival by approximately 7 to 9 months. The greatest survival benefit was observed when the renin–angiotensin–aldosterone system (RAAS) inhibitors were used in combination with agents targeting the vascular endothelial growth factor (VEGF) pathway, such as sunitinib, sorafenib, and axitinib. These findings suggest that RAAS inhibitors may exert a synergistic effect with anti-VEGF therapies by suppressing angiogenesis and slowing tumor progression. The beneficial effect of RAAS inhibitors on survival was more pronounced in patients receiving anti-VEGF therapies than in those treated with mTOR inhibitors (e.g., temsirolimus) or interferon-based regimens.

The positive association between RAAS use and improved survival appeared to be independent of baseline blood pressure levels or treatment-induced hypertension. This observation supports the hypothesis that RAAS may directly influence tumor biology, potentially through inhibition of metastasis and reduction in tumor vascularization.

In the present cohort, descriptive analyses were conducted to evaluate the distribution of these medication classes within the cohort. Due to the limited sample size, comparisons between individual drug classes were considered exploratory. After adjustment using a propensity score approach, antihypertensive therapy remained associated with improved overall survival, although the magnitude of the association was attenuated compared with the primary multivariable model. This finding suggests that baseline imbalances contributed to the observed survival difference, but do not fully explain it.

But increasing numbers of antihypertensive medications were not associated with tumor characteristics or survival outcomes. However, patients receiving more intensive antihypertensive treatment were less likely to receive later lines of systemic therapy, which may reflect a greater comorbidity burden or increased clinical frailty influencing treatment decisions rather than a direct pharmacologic effect of antihypertensive agents.

Additionally, patients receiving antihypertensive therapy may undergo more frequent medical follow-up and imaging examinations, which could contribute to earlier detection of renal tumors; however, no association between antihypertensive therapy use and stage at diagnosis was observed in this cohort.

The high proportion of stage IV disease in this cohort likely reflects referral patterns of a tertiary oncology center, where patients with advanced or metastatic RCC are more frequently managed than those with incidentally detected localized tumors.

Overall, the findings of this study support the hypothesis that antihypertensive therapy use is associated with improved survival among patients with RCC but do not demonstrate an association between AHT exposure and tumor characteristics at diagnosis. The magnitude of the observed association may reflect residual confounding and should be interpreted cautiously.

Given the observational nature of the study, these results should be interpreted as hypothesis-generating rather than evidence of a causal relationship.

## 5. Limitations

This study has several limitations that should be considered when interpreting the results. First, its retrospective single-center design introduces the possibility of selection bias and limits the generalizability of the findings to broader patient populations. Because treatment decisions and follow-up strategies were determined according to institutional practice patterns rather than predefined study protocols, unmeasured confounding variables may have influenced both antihypertensive therapy exposure and survival outcomes.

Second, although multivariable adjustment and propensity score-based analyses were performed to reduce confounding by indication, residual confounding cannot be excluded. In particular, antihypertensive therapy exposure overlapped completely with hypertension status in this cohort, making it difficult to distinguish the independent effects of hypertension itself from those of antihypertensive medications. Differences in baseline characteristics between treatment groups, including comorbidity burden and likelihood of undergoing surgical treatment, may therefore have contributed to the observed survival differences.

Third, the relatively small sample size limited statistical power for subgroup analyses, particularly comparisons between individual antihypertensive drug classes. Although descriptive analyses of antihypertensive medication categories were performed, the study was not adequately powered to evaluate class-specific survival effects or potential differential biological mechanisms among renin–angiotensin system inhibitors, calcium channel blockers, beta-blockers, and diuretics.

Fourth, detailed information regarding duration of antihypertensive therapy, medication adherence, dose intensity, and treatment modifications during follow-up was not available. Because antihypertensive exposure was defined at the time of diagnosis, longitudinal changes in treatment patterns could not be incorporated into the survival models, potentially introducing exposure misclassification.

Finally, survival analyses were restricted to patients with complete time-to-event data. Although vital status information was available for the full cohort, incomplete date information prevented inclusion of all deaths in Kaplan–Meier and Cox regression analyses. However, baseline characteristics of excluded patients did not differ substantially from those included, suggesting that the risk of selection bias related to missing time-to-event information was limited.

## 6. Conclusions

In this retrospective cohort of patients with renal cell carcinoma, antihypertensive therapy use at diagnosis was associated with improved overall survival after adjustment for established prognostic factors and propensity score-based weighting. Antihypertensive therapy use was not associated with tumor size, stage at diagnosis, or metastasis status, suggesting that the observed survival advantage was unlikely to reflect differences in baseline tumor burden.

Because antihypertensive therapy exposure overlapped completely with hypertension status and baseline clinical characteristics differed between treatment groups, the observed association should be interpreted cautiously. Residual confounding and treatment-selection bias cannot be excluded, and the findings do not establish a causal relationship between antihypertensive therapy use and improved survival outcomes.

These results support the hypothesis that antihypertensive therapy may be associated with survival differences in patients with renal cell carcinoma and highlight the need for larger prospective multicenter studies with detailed longitudinal treatment data to clarify the independent prognostic impact of antihypertensive medications in this population.

## Figures and Tables

**Table 1:** Baseline demographic, clinical, tumor characteristics, and treatment patterns according to antihypertensive therapy use.

Variable	Total (n = 99)	No AHT (n = 34)	AHT (n = 65)	*p* Value
Age (years), mean ± SD	59.5 ± 10.9	54.8 ± 12.3	61.9 ± 9.2	0.001
Male sex, n (%)	72 (72.7)	27 (79.4)	45 (69.2)	0.300
Smoking, n (%)	61 (61.0)	28 (82.4)	33 (50.8)	0.002
Alcohol use, n (%)	4 (4.0)	2 (5.9)	2 (3.1)	0.603
Additional comorbidity, n (%)	79 (79.0)	18 (52.9)	61 (93.8)	0.001
Clear cell histology, n (%)	82 (82.8)	29 (85.3)	53 (81.5)	0.395
Stage IV disease at diagnosis, n (%)	65 (65.7)	26 (76.5)	39 (60.0)	0.447
Tumor size (mm), mean ± SD	79.6 ± 34.6	79.5 ± 35.2	79.7 ± 34.5	1.000
Nodal involvement (N1), n (%)	40 (40.0)	19 (55.9)	20 (30.8)	0.020
Metastatic disease (M1), n (%)	64 (64.0)	26 (76.5)	37 (56.9)	0.062
Surgery performed, n (%)	53 (53.0)	12 (35.3)	41 (63.1)	0.011
First-line systemic therapy, n (%)	99 (99.0)	34 (100)	65 (100)	1.000
Second-line systemic therapy, n (%)	72 (72.7)	18 (52.9)	54 (83.1)	0.070
Third-line systemic therapy, n (%)	47 (47.5)	9 (26.5)	38 (58.5)	0.032
Fourth-line systemic therapy, n (%)	39 (39.4)	8 (23.5)	31 (47.7)	0.140
Deceased at last follow-up, n (%)	62 (62.6)	24 (70.6)	38 (58.5)	0.105

Values are presented as mean ± standard deviation (SD) for continuous variables and number (percentage) for categorical variables. Comparisons between patients receiving antihypertensive therapy (AHT) and those not receiving AHT were performed using independent-samples *t*-tests for continuous variables and χ^2^ or Fisher’s exact tests for categorical variables, as appropriate. All statistical tests were two-sided, and *p* < 0.05 was considered statistically significant. Tumor size was measured at the time of diagnosis. Stage was assigned according to the TNM classification at diagnosis. Systemic treatment lines refer to sequential administration of systemic therapy during follow-up. Abbreviations: AHT, antihypertensive therapy; SD, standard deviation; N1, regional lymph node involvement; M1, distant metastasis.

**Table 2:** Overall survival according to antihypertensive therapy use.

Variable	Events/Total	Median OS (Months)	Log-Rank *p*
No AHT	14/33	17.1	<0.001
AHT	17/64	112.2	

Overall survival (OS) was calculated from the date of renal cell carcinoma diagnosis to death from any cause or last follow-up. Patients alive at last follow-up were censored. Survival distributions were estimated using the Kaplan–Meier method and compared between groups using the log-rank test. Abbreviations: AHT, antihypertensive therapy; OS, overall survival.

**Table 3:** Multivariable Cox proportional hazards regression analysis for overall survival.

Variable	Hazard Ratio (HR)	95% CI	*p* Value
Antihypertensive therapy	0.26	0.13–0.50	<0.001
Age (per year)	1.02	0.98–1.05	0.308
IMDC score	1.92	1.41–2.61	<0.001
Surgery	0.16	0.06–0.45	0.001
Nodal involvement	1.19	0.58–2.43	0.630
Metastasis	1.04	0.34–3.20	0.944

Hazard ratios (HRs) and 95% confidence intervals (CIs) were estimated using multivariable Cox proportional hazards regression analysis. The final model included antihypertensive therapy, age, IMDC score, surgery, nodal involvement, and metastasis status. Stage at diagnosis was excluded from the final model due to conceptual overlap with metastasis status and IMDC risk. Abbreviations: HR, hazard ratio; CI, confidence interval; IMDC, International Metastatic Renal Cell Carcinoma Database Consortium.

## Data Availability

The data presented in this study are available on request from the corresponding author.
